# Pathological Microenvironment‐Remodeling Nanoparticles to Alleviate Liver Fibrosis: Reversing Hepatocytes‐Hepatic Stellate Cells Malignant Crosstalk

**DOI:** 10.1002/advs.202408898

**Published:** 2024-10-28

**Authors:** Ling‐Feng Zhang, Wen‐Qi Deng, Xing‐Huan Wang, Qing‐Wen Huang, Su‐Qing Liang, Ze‐Quan Ding, Liang Qi, Yi Wang, Tian‐Jiao Zhou, Lei Xing, Jai‐Woo Lee, Yu‐Kyoung Oh, Hu‐Lin Jiang

**Affiliations:** ^1^ State Key Laboratory of Natural Medicines Department of Pharmaceutics China Pharmaceutical University Nanjing 210009 China; ^2^ Department of Pediatric Surgery Children's Hospital of Nanjing Medical University 72 Guangzhou Road Nanjing Jiangsu 210000 China; ^3^ Department of Endocrinology Zhongda Hospital School of Medicine Southeast University Nanjing 210009 China; ^4^ College of Pharmacy and Research Institute of Pharmaceutical Sciences Seoul National University Seoul 08826 South Korea; ^5^ College of Pharmacy Yanbian University Yanji 133002 China; ^6^ Jiangsu Key Laboratory of Druggability of Biopharmaceuticals China Pharmaceutical University Nanjing 210009 China; ^7^ Department of Precision Medicine School of Medicine Sungkyunkwan University Suwon 16419 South Korea

**Keywords:** cellular crosstalk, chemogene therapy, hepatic stellate cell, hepatocyte, liver fibrosis

## Abstract

During the onset and malignant development of liver fibrosis, the pernicious interplay between damaged hepatocytes and activated hepatic stellate cells (HSCs) induce a self‐perpetuating vicious cycle, deteriorating fibrosis progression and posing a grave threat to public health. The secretions released by damaged hepatocytes and activated HSCs interact through autocrine or paracrine mechanisms, involving multiple signaling pathways. This interaction creates a harsh microenvironment and weakens the therapeutic efficacy of single‐cell‐centric drugs. Herein, a malignant crosstalk‐blocking strategy is prompted to remodel vicious cellular interplay and reverse pathological microenvironment to put an end to liver fibrosis. Collagenases modified, bardoxolone and siTGF‐β co‐delivered nanoparticles (C‐NPs/BT) are designed to penetrate the deposited collagen barriers and further regulate the cellular interactions through upregulating anti‐oxidative stress capacity and eliminating the pro‐fibrogenic effects of TGF‐β. The C‐NPs/BT shows successful remodeling of vicious cellular crosstalk and significant disease regression in animal models. This study presents an innovative strategy to modulate cellular interactions for enhanced anti‐fibrotic therapy and suggests a promising approach for treating other chronic liver diseases.

## Graphical abstract

The manuscript promptes an innovative strategy to modulate cellular interactions for enhanced anti‐fibrotic therapy and suggests a promising approach for treating other chronic liver diseases. Collagenases modified, bardoxolone and siTGF‐β co‐delivered nanoparticles (C‐NPs/BT) show successful remodeling of vicious cellular crosstalk and significant disease regression in animal models.

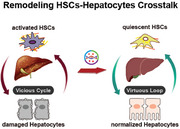

## 1 Introduction

Liver fibrosis is an uncontrollable wound‐healing response to repeated chronic liver injury.^[^ ^1^ ^]^ It is characterized by persistent hepatocyte necrosis, excessive proliferation and activation of hepatic stellate cells (HSCs), and continuous extracellular matrix (ECM) deposition.^[^ ^2^, ^3^ ^]^ Moreover, liver fibrosis has a strong relationship with liver cancer and is an unavoidable stage in the progression of various chronic injuries to end‐stage fatal liver diseases such as cirrhosis and liver cancer, evolving as a severe public health problem worldwide.^[^ ^4^ ^]^ Fortunately, clinical studies have shown that unlike cirrhosis, liver fibrosis is dynamic and reversible, presenting significant therapeutic potential.^[^ ^5^ ^]^ If the process of liver fibrosis can be controlled reasonably and effectively, it can not only maintain liver function and control disease progression but also reduce the prevalence and mortality of chronic liver disease to a certain extent.^[^ ^6^ ^]^ Therefore, effective treatment of liver fibrosis is significant for the prevention and intervention of various chronic or fatal liver diseases.

Till now, HSCs‐targeting therapies have been a primary focus since HSCs are central to liver fibrosis.^[^ ^7^ ^]^ Due to off‐target toxicity and delivery barriers, scientists have developed specific delivery systems to inhibit HSCs activation and induce HSCs senescence using targeting moieties such as hyaluronic acid, vitamin A, and chondroitin sulfate.^[^ ^8^, ^9^, ^10^ ^]^ Moreover, modifying collagenases to pass through ECM barriers and modulating liver sinusoids fenestrae to promote deep drug delivery have been shown to increase drug accumulation in fibrotic nidus and enhance the therapeutic effect.^[^ ^11^, ^12^ ^]^ However, limited by the complex pathological microenvironment that is constructed by various signaling pathways and multiple injured cells, the previous studies achieved moderate therapeutic efficiency. Therefore, a deeper understanding of the pathological mechanisms and the development of more effective therapies are urgently needed to treat or even reverse liver fibrosis.^[^ ^13^, ^14^ ^]^

In our previous studies, we have proved that synergetic regulation of cellular crosstalk and interruption of vicious interplay could improve pathological microenvironment and enhance therapeutic effect, further demonstrating the applicability of HSCs‐beyond therapy.^[^ ^15^, ^16^ ^]^ Notably, hepatocytes, the most abundant cells in the liver, also play a crucial role in the initiation and development of liver fibrogenesis.^[^ ^17^ ^]^ In response to various injuries (such as excessive alcohol, toxic substances, metabolic disorders, and viral infections), the sharply increased oxidative stress injures hepatocytes, leading to necrosis or apoptosis by increasing mitochondrial permeability. Damaged hepatocytes release a large number of signaling molecules (hedgehog, nucleotides, and free fatty acids) and produce large amounts of reactive oxygen species (ROS), which stimulate the activation of quiescent HSCs.^[^ ^18^, ^19^, ^20^ ^]^ The uncontrollably generated ROS also upregulates the expression of key genes related to collagen formation, such as type I collagen, monocyte chemoattractant protein 1, and tissue inhibitor of metalloproteinase‐1, leading to abnormal collagen deposition.^[^ ^21^ ^]^ Moreover, the excessive collagen secreted by activated HSCs would increase mechanical tension, affect the morphology and function of hepatocytes, and further aggravate hepatocytes necrosis.^[^ ^22^ ^]^ It is important to note that the malignant relationship between damaged hepatocytes and activated HSCs would create a pathological microenvironment, exacerbate cellular injury, and cause repeated stimulation, finally transforming into an uncontrollable and fatal disease.

In addition, transforming growth factor‐β (TGF‐β) is widely regarded as the most effective pro‐fibrotic cytokine.^[^ ^23^ ^]^ TGF‐β would induce the phosphorylation of drosophila mothers against decapentaplegic protein (SMAD) by binding to type I receptors, while activation of SMAD3 promotes the transcription of type I and type III collagen. Concurrently, TGF‐β would activate mitogen‐activating protein kinase signaling pathways, promoting HSCs activation. TGF‐β also inhibits hepatocyte proliferation and hinders liver regeneration.^[^ ^24^ ^]^ Therefore, inhibiting TGF‐β can reduce collagen fiber synthesis, inhibit HSCs activation, and promote hepatocyte regeneration. This suggests a potential synergetic therapeutic effect when combined with modulation of oxidative stress to reverse the malignant crosstalk between damaged hepatocytes and malignant HSCs, and to remodel the liver microenvironment.

**Scheme 1:**
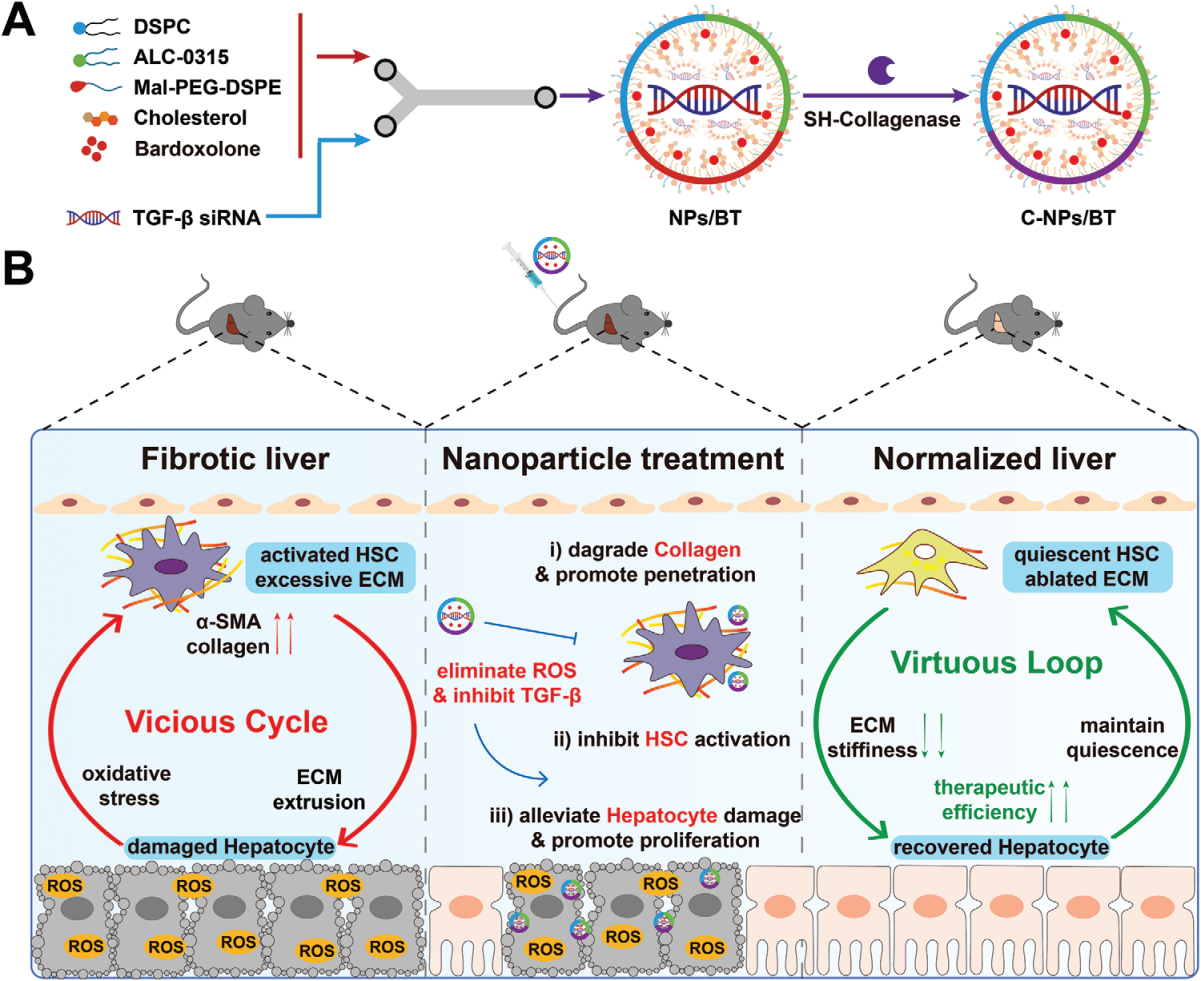
The preparation and in vivo mechanism of C‐NPs/BT. Briefly, C‐NPs/BT relieve hepatocytes damage and inhibit HSC activation by eliminating oxidative stress and silencing TGF‐β signaling pathway synergistically, thereby interrupting the malignant cellular interactions. C‐NPs/BT would significantly remodel the pathological microenvironment and promote the resolution of liver fibrosis.

Based on our previous studies and the interplay between these two types of pathological cells, we hypothesized that blocking the malignant interaction and improving the pathological microenvironment by synergistic modulating oxidative stress and the TGF‐β signaling pathway could reverse the vicious cycle, normalize cellular interactions, and lead to complete disease resolution. Herein, a malignant crosstalk‐blocking strategy was prompted by using chemogene therapy to remodel vicious cellular interplay and reverse pathological microenvironment to terminate liver fibrosis. Briefly, collagenases modified, bardoxolone, and siTGF‐β co‐encapsulated nanoparticles, nominated as C‐NPs/BT, were constructed. The grafted collagenases are expected to help penetrate ECM barriers and promote cellular uptake. Bardoxolone, a potent antioxidant, is intended to repair damaged hepatocytes, relieve oxidative stress, and reduce HSC activation.^[^ ^25^ ^]^ The loaded siTGF‐β is expected to prevent further HSC activation and promote hepatocyte proliferation using RNA interference strategies. By applying this integrative and straightforward strategy, the malignant interplay between damaged hepatocytes and activated HSCs would be interrupted, fostering a healthy liver microenvironment. This creates a positive feedback loop that amplifies the therapeutic effect (**Scheme** **1**). This work pioneers the use of pathological microenvironment remodeling therapy to break the vicious cycle of liver fibrosis, offering a novel perspective for liver fibrosis treatment.

## 2 Results and Discussion

### 2.1 Malignant Crosstalk Between Hepatocytes and HSCs in Human Fibrotic Liver

**Figure 1:**
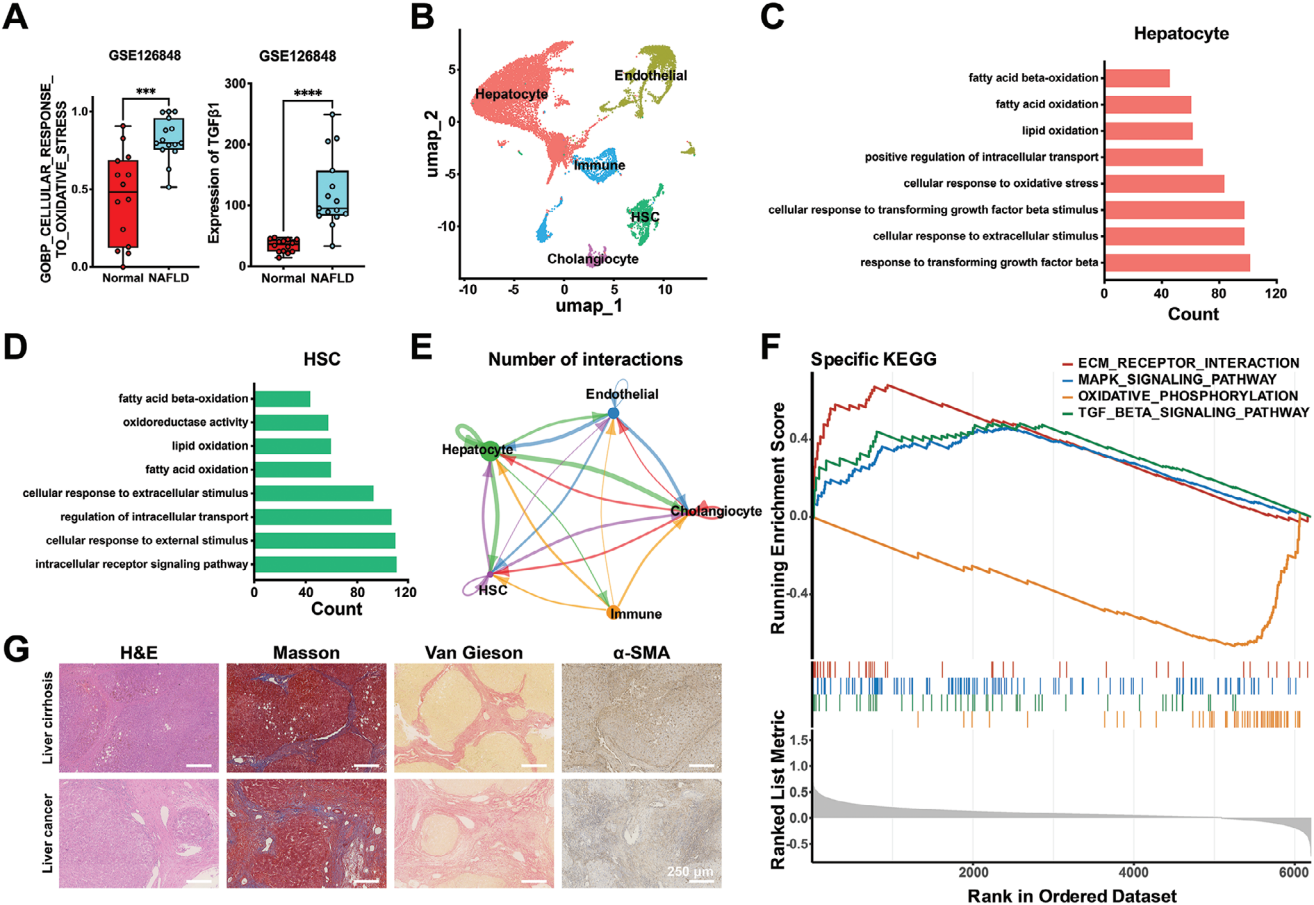
The vicious crosstalk between hepatocytes and HSCs in human fibrotic liver. A) Bulk‐sequence analysis of cellular response to oxidative stress and TGFβ1 expression. B) UMAP plot showing the distribution of five major cell types. C) Signaling pathway enriched in hepatocyte. D) Signaling pathway enriched in HSC. E) Cell chat analysis of interactions among the five major cell types. F) KEGG analysis of gene enrichment related to these pathways. G) Representative images of tissue sections of patients's liver, showing H&E, Masson, Van Gieson, and α‐SMA staining. Data was expressed as mean ± SD. ^***^ *p* < 0.001, ^****^ *p* < 0.0001.

Hepatocytes and HSCs are the two main components of hepatic cells. While hepatocytes are the most numerous, HSCs serve as the central hub during liver fibrogenesis. To obtain a deeper understanding of the pathological microenvironment in fibrotic liver, primary data from GSE126848 was re‐analyzed. The results showed increased levels of oxidative stress, TGFβ1, and fibroblast activation in NAFLD patients (**Figure** **1**A; Figure S1, Supporting Information).^[^ ^26^ ^]^ For further evaluation of the importance of hepatocytes and HSCs, we then analyzed primary data from NCBI database (GSE212837).^[^ ^27^ ^]^ The extracted cells were divided into five groups: hepatocyte, HSC, cholangiocytes, immune cells, and endothelial cells (Figure 1B). Oxidative stress and TGF‐β‐related signaling pathways were significantly upregulated in fibrotic liver (Figure 1C,D). Cell chat analysis revealed a sharp increase in cellular interactions between hepatocytes and HSCs during fibrogenesis (Figure 1E). Moreover, KEGG analysis showed gene enrichment related to these pathways, highlighting the significance of modulating oxidative stress and TGF‐β signaling in damaged hepatocytes and activated HSCs (Figure 1F). Furthermore, analysis of human tissue from patients with liver cirrhosis or liver cancer revealed significant findings. H&E staining showed damaged hepatocytes and disrupted tissue structure, while Masson and Van Gieson staining exhibited excessive ECM deposition gradually replacing liver parenchyma. α‐SMA staining revealed high activation of HSCs in fibrotic liver (Figure 1G).

### 2.2 Preparation, Characterization and Biosafety of C‐NPs/BT

**Figure 2:**
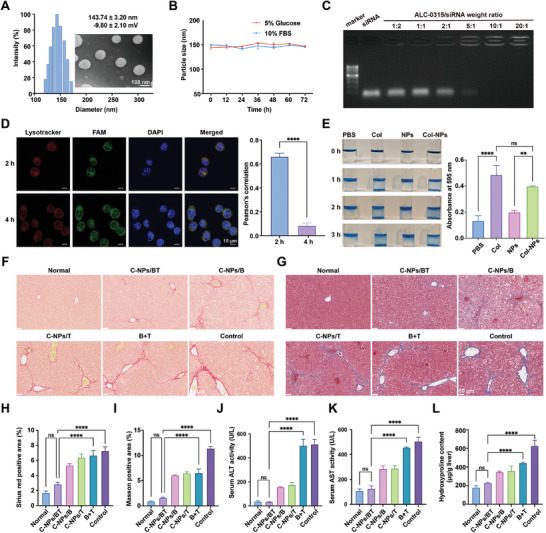
Characterization and in vivo anti‐fibrotic effects of C‐NPs/BT. A) Size distribution and TEM images of C‐NPs/BT. Scale bar = 100 nm B) Size variation in 5% glucose and 10% FBS 1640 culture medium over 72 h. C) Agarose gel electrophoresis of ALC‐0315/siRNA at various weight ratios. D) Lysosomal escape assay on HSC‐T6 cells. Scale bar = 10 nm E) Visualized collagen degradation experiments and quantification (*n* = 3). F) Representative images of Sirius Red staining. Scale bar = 50 µm G) Representative images of Masson staining. Scale bar = 50 µm H) Semi‐quantification results of Sirius Red positive area (*n* = 3). I) Semi‐quantification results of Masson positive area (*n* = 3). J) Serum ALT activity (*n* = 3). K) Serum AST activity (*n* = 3). L) Tissue hydroxyproline content (*n* = 3). C‐NPs/BT: nanoparticles modified with collagenases and loaded with bardoxolone and siTGF‐β; C‐NPs/B: nanoparticles modified with collagenases and loaded with bardoxolone; C‐NPs/T: nanoparticles modified with collagenases and loaded with siTGF‐β; B+T: free bardoxolone and siTGF‐β. Data was expressed as mean ± SD. No significant difference (ns): *p* > 0.05, ^**^ *p* < 0.01, and ^****^ *p* < 0.0001.

To reverse the malignant crosstalk between damaged hepatocytes and activated HSCs by synergistically modulating related cell injury, C‐NPs/BT were prepared using T‐tubes for chemogene therapy. The optimal molar ratio of ALC‐0315: Cholesterol: DSPC: DSPE‐PEG‐Mal was determined to be 45: 40: 10: 5 (Table S1, Supporting Information). TEM images revealed that C‐NPs/BT had a spherical and uniform appearance (**Figure** **2**A). The average particle size was 143.74 ± 3.20 nm, and the zeta potential was ‐9.80 ± 2.10 mV. C‐NPs/BT exhibited good stability in 10% FBS or 5% glucose, with no significant changes in particle size over 72 h (Figure 2B). The drug loading and encapsulation efficiency were measured to be 3.38 ± 0.08% and 74.00 ± 4.91%, respectively. The agarose gel retardation study showed that the cationic lipids ALC‐0315 and siRNA were completely complexed at a weight ratio of 10:1 (Figure 2C).

To verify the gene‐protecting function of the nanoparticles, a lysosomal escape assay was performed on HSC‐T6 cells. The results showed that the cationic lipids facilitated lysosomal escape through the nephew sponge effect (Figure 2D). Collagenases were modified via click chemistry reactions between the maleimide group and the thiol group. Visual inspection using Prussian blue revealed that C‐NPs could ablate deposited collagen and achieve deeper penetration (Figure 2E).

Due to the potential toxicity limiting the application of nano‐delivery system, the biosafety of C‐NPs/BT was tested on cells and healthy mice. As shown in Figure S2 (Supporting Information), C‐NPs/BT showed no obvious cell toxicity on three dominant hepatic cells, hepatocytes (L02), HSCs (HSC‐T6), and macrophages (RAW 264.7) over 24 and 48 h, with cell viability above 80%. The blood compatibility of the nanoparticles was also tested. Similar to PBS, C‐NPs/BT,C‐NPs/B, C‐NPs/T, and B+T (bardoxolone and siTGF‐β) showed good blood compatibility (Figure S3, Supporting Information).

Next, the acute toxicity of nanoparticles was evaluated. Different preparations were injected intravenously into healthy mice for three continuous days and the blood sample was analyzed. There was no significant difference in ALT and AST levels between the C‐NPs/BT group and the PBS group, demonstrating biosafety in liver (Figure S4, Supporting Information). Moreover, H&E staining revealed no significant inflammation in major tissues, such as liver, spleen, kidney, lung, and heart after repeated administration, further confirming the biosafety of C‐NPs (Figure S5, Supporting Information).

### 2.3 Anti‐fibrotic Effects of C‐NPs/BT In Vivo

To explore the therapeutic effect of C‐NPs/BT in vivo, a carbon tetrachloride (CCl_4_)‐induced liver fibrosis model was established. Mice were divided into six groups and were injected with CCl_4_ intraperitoneally twice a week for six weeks. The groups included normal mice and fibrotic mice treated with C‐NPs/BT,C‐NPs/B, C‐NPs/T, B+T, and PBS (Control group) to compare the efficacy (Figure S6, Supporting Information). Sirius Red and Masson staining showed reduced ECM deposition in the C‐NPs/BT treated group, which was significantly different from the Control group. (Figure 2F,G) The semi‐quantification further confirmed the substantial ECM ablation and fibrosis resolution in the C‐NPs/BT treated group. (Figure 2H,I). In contrast, heavy ECM deposition was observed in the C‐NPs/B, C‐NPs/T, and B + T groups, demonstrating the limited effect of single‐target therapy for hepatocytes or HSCs. Serum ALT and AST activity indicated that chemogene therapy with C‐NPs/BT could reduce hepatocyte injury and protect liver function, mainly by remodeling the malignant interplay between damaged hepatocytes and activated HSCs (Figures 2J,K).

As a main component of ECM, hydroxyproline content indicates the severity of fibrosis. The hydroxyproline content in the C‐NPs/BT treated group significantly decreased and was comparable to that of normal group, unlike the B+T treated group (Figure 2L). Moreover, the H&E stained sections of other major organs (spleen, kidneys, lung, and heart) showed no obvious difference from those of normal mice, suggesting the safety of this chemogene therapy (Figure S7, Supporting Information). These results comprehensively support the potent therapeutic effect of this combined therapy, suggesting its potential for the clinical application in remodeling the harmful crosstalk between damaged hepatocytes and activated HSCs.

### 2.4 Hepatocytes Injury Relieving Effects and Mechanism Verification

**Figure 3:**
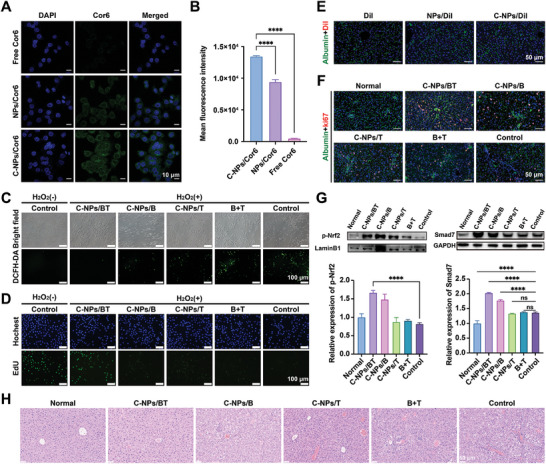
The effect of C‐NPs/BT on hepatocytes in vitro and in vivo. A) Representative CLSM images of L02 cells after 2 h treatment. Scale bar = 10 µm B) Mean fluorescence intensity measured by flow cytometry (*n* = 3). C) Representative images using DCFH‐DA probe to test ROS levels. Scale bar = 100 µm D) Representative images of EdU staining. Scale bar = 100 µm E) Representative images of albumin immunofluorescent‐staining (blue: nucleus; green: albumin; red: DiI). Scale bar = 50 µm F) Representative images of ki67 staining (blue: nucleus; green: albumin; red: ki67). Scale bar = 50 µm G) Western blot and semi‐quantification for p‐Nrf2 and Smad7 (*n* = 3). H) Representative images of H&E staining. Scale bar = 50 µm Data was expressed as mean ± SD. No significant difference (ns): P > 0.05 and ^****^ *p* < 0.0001.

Hepatocytes injury and increased membrane permeability are inevitable problems in liver fibrosis caused by various etiologies. For further exploration of the effects of C‐NPs/BT on damaged hepatocytes, the cellular uptake was first investigated. To simulate the deposited ECM in the Disse space, collagen I (150 µg mL^−1^) was pre‐layered on L02 cells for 5 h. The fluorescence in the free drug (coumarin 6, Cor6) treated group was almost invisible, suggesting minimal uptake by hepatocytes (**Figure** **3**A). In contrast, the NPs/Cor6 treated group showed slightly higher fluorescence, while those treated with C‐NPs/Cor6 was much brighter green fluorescence. This increase is mainly due to the modification with collagenases, which could ablate deposited collagen and enhance uptake. Flow cytometry quantitation showed similar results (Figure 3B). The DCFH‐DA probe demonstrated that, compared to other nanoparticles, the final preparation effectively downregulated the level of oxidative stress (Figure 3C). Moreover, to assess the ability of C‐NPs/BT to promote hepatocytes proliferation, Edu staining was applied (Figure 3D). The bright green fluorescence in C‐NPs/BT treated group indicated that the preparation could mitigate the cell damage caused by H_2_O_2_ and maintain cell activity.

For lipid nanoparticles, the distribution is mainly dependent on the compositions and the proteins absorbed. C‐NPs were composed of commonly‐used materials, namely DSPC, ALC‐0315, DSPE‐PEG‐Mal, and cholesterol. In our previous study and other references, the biodistribution of such kind of lipid nanoparticles is mainly distribute to the liver and the modification of collagenase would enhance liver accumulation by penetrating the collagen barrier.^[^ ^8^, ^11^, ^28^ ^]^ Therefore, following the 3R principle of animal experiments, we omitted the in vivo biodistribution of nanoparticles in this project. To test whether C‐NPs/BT could fully function in vivo, the biodistribution in liver was then examined. Albumin was used as the marker of hepatocytes. As shown in Figure 3E, the red fluorescence of C‐NPs/DiI was significantly brighter than that of NPs/DiI and free DiI. This observation could be attributed to the fact that collagenase could ablate excessive collagen, thereby promoting deeper accumulation. Next, the liver sections of CCl_4_‐induced fibrotic mice were stained with ki67 (Figure 3F). The red fluorescence in the treated groups showed enhanced hepatocytes proliferation compared to the control group. Moreover, the combination of bardoxolone and siTGF‐β (C‐NPs/BT) exhibited more red fluorescence than C‐NPs/B and C‐NPs/T treated group. This result suggests that eliminating oxidative stress and inhibiting TGF‐β could synergistically promote cell viability and growth.

Additionally, the molecular mechanisms were verified by measuring related protein levels. Western blot analysis showed that the expression of phosphorylated nuclear factor E2‐related factor 2 (p‐Nrf2), Smad7, and heme oxygenase 1 (HO‐1) in the C‐NPs/BT treated group was significantly upregulated in hepatocytes and fibrotic mice (Figure 3G; Figure S8, Supporting Information). This is primarily because bardoxolone could stimulate Nrf2, an important transcription factor that regulates antioxidant responses in hepatocytes. Under physiological conditions, Nrf2 forms a complex with Keap1 and is degraded by ubiquitination. In response to oxidative stress, Nrf2 separates from Keap1, accumulates in the cytoplasm, and translocates to the nucleus to initiate transcription of antioxidant protective genes. Upregulated Nrf2 would further upregulate HO‐1 transcription to eliminate excessive ROS. Bardoxolone also increases the level of Smad7 and reduced the expression of TGF‐β, achieving the anti‐fibrotic effect. It was observed that the expression of Smad7 in the control group was higher than that in the normal group, which might be explained as compensatory increase. Compared to normal mice, those treated with PBS exhibited swollen hepatocytes and destroyed tissue structures (Figure 3H). However, the C‐NPs/BT treated group showed recovered hepatocytes and less inflammatory cell infiltration than C‐NPs/B, C‐NPs/T, B+T treated groups, preliminarily proving the great effect of this chemogene therapy to remodel the vicious crosstalk between damaged hepatocytes and activated HSCs.

### 2.5 HSCs Activation Inhibiting Effects and Mechanism Verification

**Figure 4:**
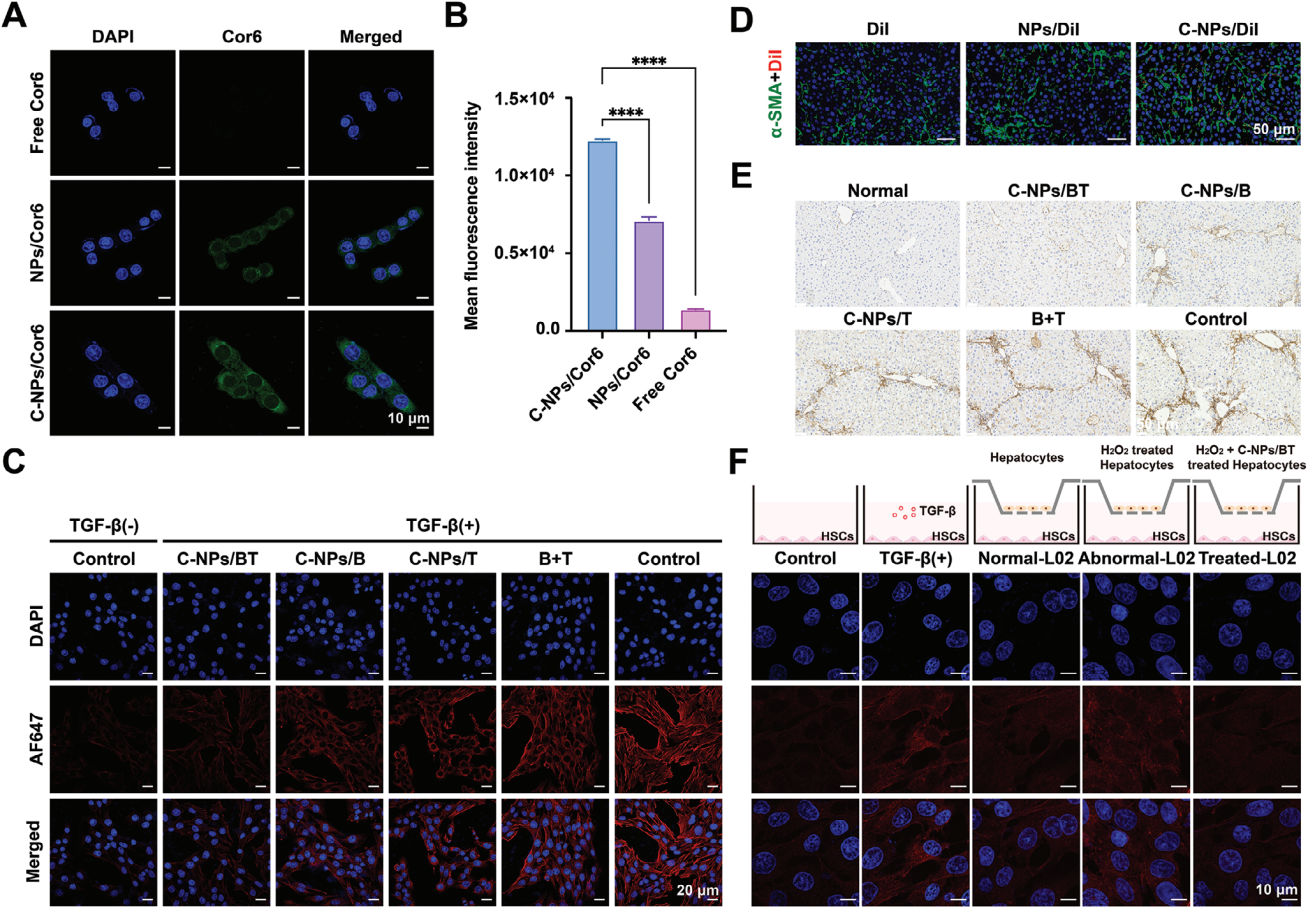
The effect of C‐NPs/BT on HSCs in vitro and in vivo. A) Representative CLSM images of HSC‐T6 cells after 2 h. Scale bar = 10 µm B) Mean fluorescence intensity measured by flow cytometry (*n* = 3). C) Representative images of α‐SMA immunofluorescent staining on HSC‐T6 cells. Scale bar = 20 µm D) Representative images of α‐SMA immunofluorescent staining in vivo. Scale bar = 50 µm E) Representative images of α‐SMA‐stained liver sections. Scale bar = 50 µm F) Representative images of α‐SMA staining in a coculture model. Scale bar = 10 µm Data was expressed as mean ± SD. ^****^ *p* < 0.0001.

During the pathological process, activated HSCs secrete higher levels of tissue inhibitors of metalloproteinases‐1 and collagen I, which lead to excessive extracellular matrix and hinder the drug delivery. The activated HSCs caused an imbalance between collagen generation and degradation, resulting in aggravated liver fibrosis. Therefore, the therapeutic effect of C‐NPs/BT in inhibiting HSC activation was further explored. Cor6 was also used as a model drug to investigate the cellular uptake of C‐NPs/BT in the presence of collagen barrier. It was demonstrated that the collagenases helped improve the uptake efficiency by about two folds, greatly higher than that of free Cor6 (**Figure** **4**A,B). Next, TGF‐β (10 ng/mL) was used to stimulate HSC activation in vitro. As shown in Figure 4C and Figure S9 (Supporting Information), the red fluorescence of α‐SMA and collagen I represents the activation degree of HSCs. After stimulation, the control group exhibited heavily deposited collagen, while the other groups could counteract its stimulating effect to a certain extent and C‐NPs/BT showed the optimal effect. C‐NPs/BT work in two ways: i) the loaded bardoxolone eliminate ROS through Nrf2‐HO‐1 pathways to inhibit HSCs phenotype transformation; ii) the loaded siTGF‐β exert gene‐silencing effect to inhibit HSCs activation.

From our preliminary study, we found that endowing nanocarriers with a proteolytic surface could ablate the collagen barrier, promote their permeation in fibrotic extracellular matrix, and finally help delivery of carriers to activated HSCs in vivo. To investigate the cellular localization of the nanoparticles, tissue immunofluorescent staining was performed. The hydrophobic fluorochrome, DiI, was intravenously administered into mice that had been treated with CCl_4_ for 4 weeks (once a day, three days), either alone or complexed with different nanoparticles. The green fluorescence of α‐SMA was highly merged with the red fluorescence of DiI in C‐NPs/DiI group, demonstrating that C‐NPs/DiI were taken up by HSCs (Figure 4D).

Moreover, α‐SMA‐stained liver sections showed that, compared to C‐NPs/B, C‐NPs/T, B+T, and the control group, C‐NPs/BT could effectively inhibit activation of HSCs and liver fibrogenesis in animal models, similar to the normal group (Figure 4E). The enhanced therapeutic effect might be due to the ability of C‐NPs/BT to relieve hepatocyte injury and promote cell proliferation, interrupting the stimulation from hepatocytes to HSCs.

### 2.6 Interruption of Vicious Interplay Between Damaged Hepatocytes and Activated HSCs

The interactions between damaged hepatocytes and activated HSCs are hypothesized to aggravate cellular damage and repeatedly stimulate HSC activation, accelerating disease progression. To investigate whether C‐NPs/BT could interrupt the vicious interplay and reverse pathological environment, a cell coculture model was established. Briefly, L02 cells were pre‐treated with H_2_O_2_ (1 mm) for 4 h and then were seeded onto the upper plate, while quiescent HSCs were seeded onto the lower plate (Figure 4F). Upon cell adhesion, the medium was replaced with fresh medium, and the coculture began. Confocal laser scanning microscopy showed that with the treatment of TGF‐β and the abnormal‐L02, HSCs were activated and secreted excessive collagen. However, Treated‐L02 treated HSCs showed almost no collagen deposition and was even lower than that of the control group, possibly because HSCs cultured with 10% FBS were partially activated.

The detrimental effect of HSCs on hepatocytes is thought to result from the collagen deposited by activated HSCs, which can cause hepatocyte injury through mechanical pressure. As liver fibrosis progresses, the nature and quantity of liver collagen gradually change. Normally ordered and loose collagen fibers become thicker, more disorganized, and increasingly interconnected. Consequently, the mechanical force in the Disse space would increase significantly. It is proposed that after the incubation of C‐NPs/BT, the modified collagenases could ablate deposited collagen and induce HSCs to adopt a collagen‐degrading phenotype, thus alleviating hepatocytes injury. Moreover, the re‐quiescent HSCs would reduce oxidative stress level, further mitigating the harmful effects on hepatocytes. We cocultured damaged hepatocytes and activated HSCs in vitro to test the efficacy of C‐NPs/BT. As shown in Figure S10 (Supporting Information), C‐NPs/BT treatment could obviously decrease the green fluorescence of H_2_O_2_ and the red fluorescence of α‐SMA, compared with PBS‐treated group. Therefore, the strategy to recover activated HSCs and damaged hepatocytes is expected to achieve greater therapeutic efficacy by remodeling cellular crosstalk and reversing the pathological microenvironment. This suggests the great clinical potential of C‐NPs/BT for treating liver fibrosis.

## 3 Conclusion

With a deeper understanding of the mechanisms of liver fibrosis, the need to go beyond the previous single‐cell‐centric therapies has become more urgent. In summary, this study proposed a malignant crosstalk‐remodeling strategy to interrupt the malignant interplay between damaged hepatocytes and activated HSCs by synergistically regulating oxidative stress and TGF‐β signaling pathway. This approach achieved promising results in a CCl_4_‐induced fibrotic mice model. After effectively interrupting the vicious cycle, it was hypothesized that a virtuous loop would gradually form, primarily because the recovered hepatocytes and re‐quiescent HSCs would benefit each other. Building on our previous study, this study offers a new approach to treat liver fibrosis that goes beyond targeting HSCs alone. Moreover, the preparation of C‐NPs/BT is simple and reproducible, enhancing the clinical feasibility and controllability.

It is worth noting that other major organs may also affect the liver. During fibrogenesis, it has been proved that at least the lung, the spleen, and the adipose tissue would affect the process of liver fibrosis. Specifically, disruption of the gut barrier and the translocation of harmful gut microbes to the liver would promote macrophages polarization, and thus aggravate fibrosis progression.^[^ ^29^ ^]^ Moreover, spleen derived‐monocytes would migrate to the liver and function as macrophages, triggering liver inflammation and finally exacerbate fibrosis.^[^ ^30^ ^]^ Besides, the lipid metabolism was disturbed in fibrotic liver and adipose tissue, and the accumulated excessive lipid would lead to vacuolization and ballooning degeneration of hepatocytes. The adipose tissue would release inflammatory factors and excessive fatty acid to the liver and therefore worsen the disease.^[^ ^31^ ^]^ Therefore, it may be a useful mean to develop multiple organ‐targeted therapy to treat liver fibrosis.

## 4 Experimental Section

### Ethics Statement

Experimentation on animals was conducted in accordance with the guidelines approved by the regional ethics committee of China Pharmaceutical University (2023‐03‐002). Human studies were performed in compliance with the ethical standards of the 1964 Declaration of Helsinki and its later amendments, and had been approved by the local Ethics Committee of Zhongda Hospital affiliated with Southeast University (Ethics number: 2018ZDSYLL070‐P01). All participants gave their informed consent prior to inclusion in the study.

### Statistical Analysis

All data were expressed as mean ± standard deviation (SD) and analyzed by Prism 10. Statistical significance was calculated using one‐way ANOVA test with the Tukey's multiple comparison analysis or two‐tailed *t*‐test and defined as p < 0.05 (ns: *p* > 0.05, ^*^ *p* < 0.05, ^**^ *p* < 0.01, ^***^ *p* < 0.001, and ^****^ *p* < 0.0001).

## Conflict of Interest

The authors declare no conflict of interest.

## Supporting information



Supporting Information

## Data Availability

The data that support the findings of this study are available from the corresponding author upon reasonable request.
