# Supplementary material for: Pathological Microenvironment‐Remodeling Nanoparticles to Alleviate Liver Fibrosis: Reversing Hepatocytes‐Hepatic Stellate Cells Malignant Crosstalk
Source: Adv Sci (Weinh). 2024 Oct 28;12(4):2408898. doi: 10.1002/advs.202408898 (PMC11775515; doi:10.1002/advs.202408898)
Supplement: Supplementary file 1 — Supporting Information [file ADVS-12-2408898-s001.docx]

**Supporting Information**

**Pathological Microenvironment Remodeling Nanoparticles** **to Alleviate Liver Fibrosis: Reverse Hepatocytes-Hepatic Stellate Cells** **Malignant Crosstalk**

Ling-Feng Zhang,^a,1^ Wen-Qi Deng,^a,1^ Xing-Huan Wang,^a,1^ Qing-Wen Huang,^a^ Su-Qing Liang,^a^ Ze-Quan Ding,^f^ Liang Qi,^g^ Yi Wang,^a^ Tian-Jiao Zhou,^a^ Lei Xing,^a^ Jai-Woo Lee,^e^ Yu-Kyoung Oh,^e^ Hu-Lin Jiang,^a, b, c, d^*

^a^ State Key Laboratory of Natural Medicines, Department of Pharmaceutics, China Pharmaceutical University, Nanjing 210009, China

^b^ College of Pharmacy, Yanbian University, Yanji 133002, China

^c^ Jiangsu Key Laboratory of Druggability of Biopharmaceuticals, China Pharmaceutical University, Nanjing 210009, China

^d^ Department of Precision Medicine, School of Medicine, Sungkyunkwan University, Suwon 16419, South Korea

^e^ College of Pharmacy and Research Institute of Pharmaceutical Sciences, Seoul National University, Seoul 08826, Korea

^f^ Department of Pediatric Surgery, Children's Hospital of Nanjing Medical University, 72 Guangzhou Road, Nanjing, 210000, Jiangsu Province, China.

^g^ Department of Endocrinology, Zhongda Hospital, School of Medicine, Southeast University, Nanjing 210009, China

*Corresponding Author

Prof. Hu-Lin Jiang, State Key Laboratory of Natural Medicines, Department of Pharmaceutics, China Pharmaceutical University, Nanjing 210009, China. E-mail: jianghulin3@gmail.com (H.L. Jiang).

**Materials and Reagents:** ALC-0315 was obtained from Med Chem Express Co., Ltd. Collagenase I was purchased from Sigma Aldrich Sigma Aldrich Shanghai Co., Ltd. Bardoxolone was purchased from Med Chem Express Co., Ltd. DSPC was purchased from AVT (Shanghai) Pharmaceutical Tech Co., Ltd. DSPE-PEG_2k_-Mal was purchased from Shanghai Aladdin Biochemical Technology Co., Ltd. Fetal bovine serum (FBS) was purchased from Thermo Fisher Scientific. DAPI, 10× PBS, BCA reagent kit, DMEM incomplete low sugar medium and DMEM incomplete high sugar medium were purchased from Jiangsu Keygen Biotech Co., Ltd. Anti-collagen I and anti-α-SMA were purchased from abcam. Hydroxyproline detection kit was purchased from Nanjing Jiancheng Technology Co., Ltd.

**Preparation of SH-Collagenase I:** 150 mg Collagenase Ⅰ and 4.13 mg dimethylamino sulfide were dissolved in 5 mL PBS, stirred at room temperature for 1 h. The dextran gel G-25 was soaked in distilled water overnight. After the upper floating debris was washed with distilled water, the bubble was removed by ultrasound, the chromatographic column was set up, and the samples were purified after equilibrium then collected the products.

**Preparation of C-NPs/BT:** ALC-0315, DSPC, cholesterol, and DSPE-PEG-Mal were dissolved in 90% ethanol in a certain proportion to form the oil term. Dissolved si-TGFβ in citric acid buffer solution with pH = 4 to form the water phase. Both were mixed into citrate buffer solution with pH = 6 by microfluidic device, and centrifuged at 5000 rpm for 3 min to dealcoholize. Wash with 200 μL PBS, add SH-Collagenase Ⅰ, and incubate overnight.

**Determination of Collagen Penetration:** 500 μL of Collagen Type I (3 mg/mL) was prelaid on 0.2% agarose gel (3mL). After placing at 37℃ for 24 h, different groups of preparations and Komas brilliant blue dye was given to each experimental group while the leakage of dye was inspected after 0, 1, 2 h of incubation at 37℃. The agarose gel was melted with heat and its absorbance at 595 nm was measured using Microplate Reader after 2 h.

**Determination of Binding Ratio of ALC-0315 and siTGF-β:** 0.2% agarose gel solution was prepared and added to the gel mold in advance. After 30 min of solidification, 8 μL marker DNA and 15 μL ALC-0315/siTGF-β mixed solution of different proportions were added from left to right into the sample hole, respectively. Gel images were photographed for subsequent analysis using gel imaging system after 100 V, 24 min of electrophoresis.

**Cellular Uptake Assay by CLSM:** Taking the uptake of L0_2_ as an example, L0_2_ was evenly spread on the confocal dish at 80,000 cells per well and cultured for 24 h. Then the medium was removed and 1 mL Col I layer (150 μg/mL) was added. After incubation for 5 h, L0_2_ was treated respectively with FBS-free medium only and FBS-free medium containing free Cor6 or C-NPs/Cor6 (the concentration of Cor6 is 0.1 μg/mL) for 1 h at 37℃ without light. Subsequently the cells were washed with PBS for 3 times and 1 mL of paraformaldehyde was added. After incubation at room temperature for 30 min, the cells were washed with PBS for 3 times. Adding 200 μL DAPI for 20 min followed by PBS washing.

**Cellular Uptake Assay by Flow Cytometry:** Taking the uptake of L0_2_ as an example, L0_2_ was evenly spread in 24-well plate at 80,000 cells per well. After incubation for 24 h, the medium was removed. L0_2_ was treated respectively with FBS-free medium only and FBS-free medium containing free Cor6 or C-NPs/Cor6 (the concentration of Cor6 is 0.1 μg/mL) for 1 h at 37℃ without light. Then the cells were washed 3 times with PBS, the supernatant was removed by centrifugation in a 1.5 mL EP tube, and 400 μL PBS was added to blow and suspend the cells. The fluorescence intensity was determined by flow cytometry.

**Oxidative Stress Assay:** L0_2_ was spread in 12-well plate at 200,000 cells per well and cultured for 24 h. After removing the medium, L0_2_ was damaged for 4 h by adding 1 mL H_2_O_2_ (1 mm, in FBS-free medium). In the presence of H_2_O_2_, B+T, C-NPs/B, C-NPs/T or C-NPs/BT were added respectively and cultured for 24 h (BARD, 300 nm; siTGF-β, 50 pm). At the same time, the non-injured group was used as negative control, and the injured group was used as positive control. Then the cells were washed twice using PBS, and 1 mL DCFH probe was added and incubated for 30 min in the dark. After the incubation, the cells were washed twice with PBS and were covered with 500 μL FBS-free medium to photograph by using inverted fluorescence microscope.

**Establishment of mice model:** Healthy and weight-balanced mice were randomly divided into 6 groups (Normal, C-NPs/BT, C-NPs/B, C-NPs/T, B+T, Control), with 10 mice in each group. The model and treatment groups were intraperitoneally injected with olive oil solution containing 25 % CCl_4_ at a dose of 2.5 mg/kg according to the body weight of the mice, and the normal group was injected with olive oil solution twice a week for 6 weeks. After 6 weeks, the treatment group was given C_BARD_ = 5 mg/kg, C_siTGF-β_ = 1 mg/kg, free drug group (B+T), single gene lipid nanoparticles (C-NPs/T), single drug lipid nanoparticles (C-NPs/B), double drug lipid nanoparticles (C-NPs/BT). Normal and Control groups were given the same amount of PBS solution as control, twice a week for two weeks.


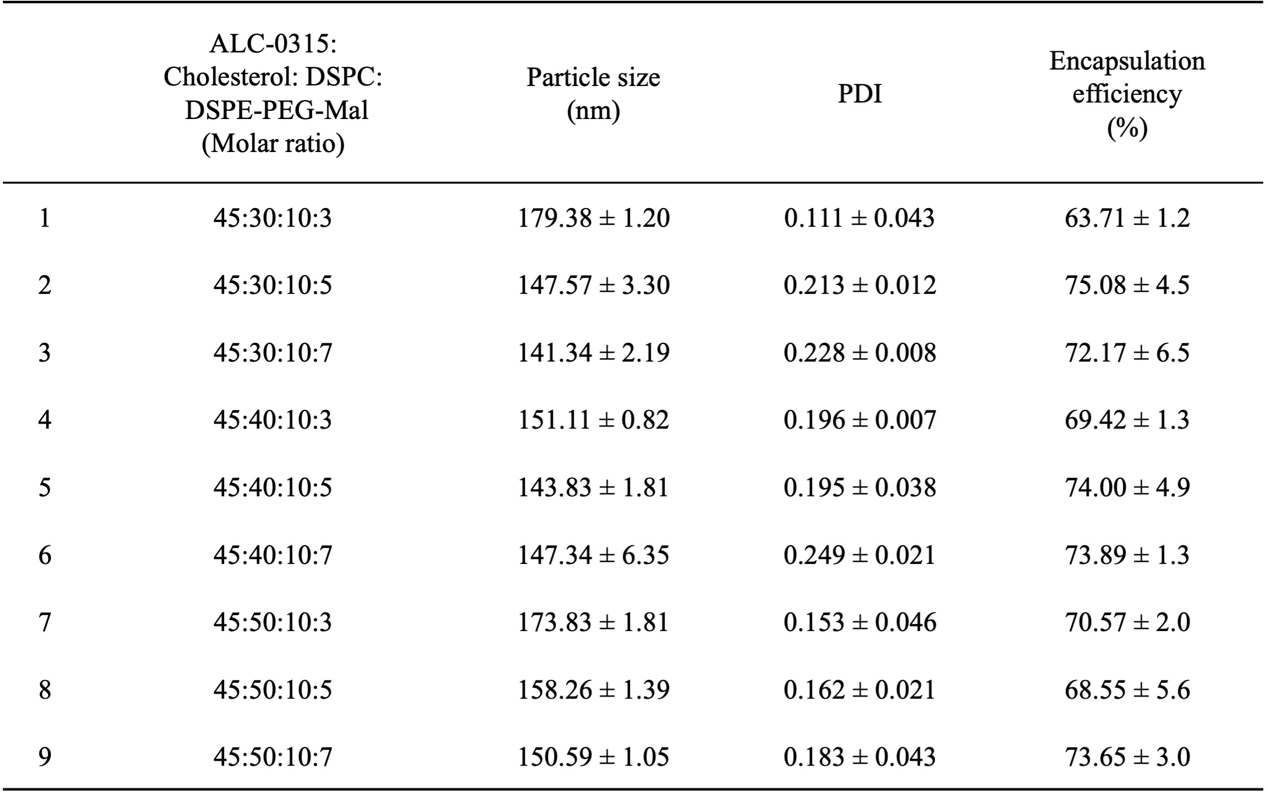


**Table S1.** Orthogonal experiment to screen optimal prescription.


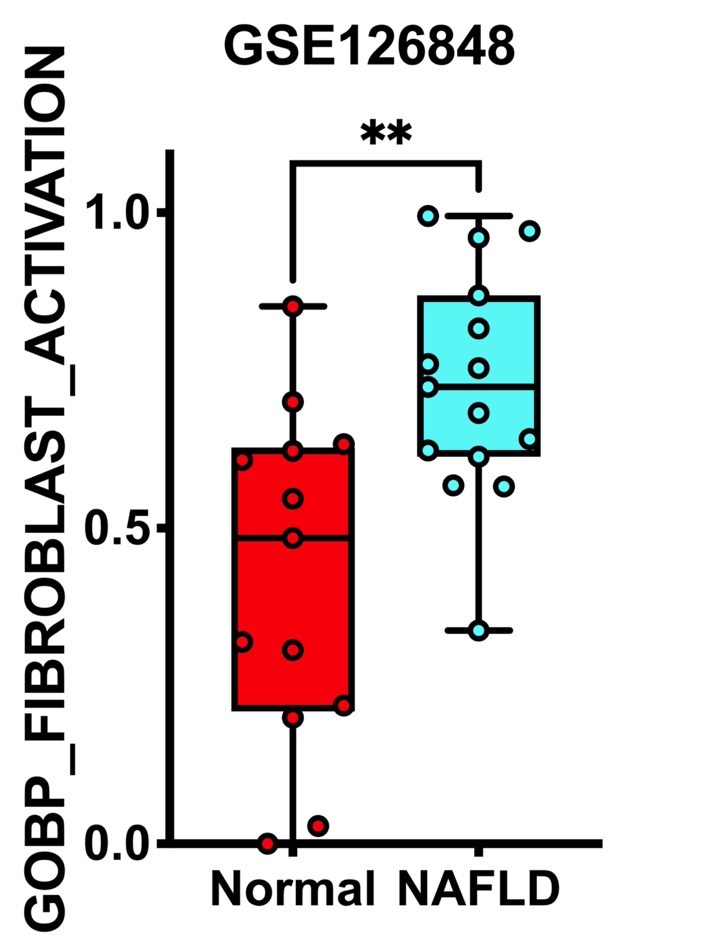


**Figure S1.** Bulk-sequence analysis of fibroblast activation via ssGSEA. Data was expressed as mean ± SD. **P < 0.01.


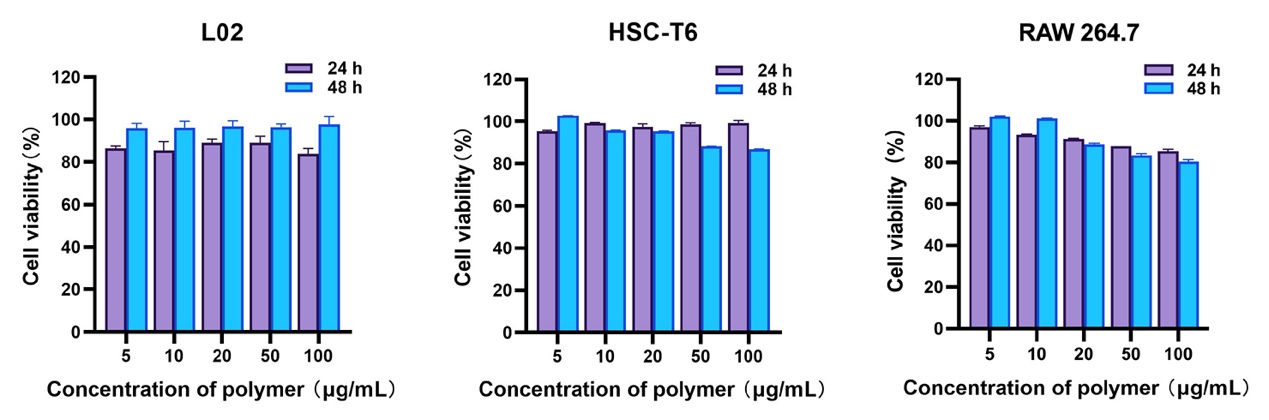


**Figure S2.** Cellular toxicity of C-NPs/BT on L02, HSC-T6 and RAW 264.7 cells at 24 and 48 h (n = 3). Data was expressed as mean ± SD.


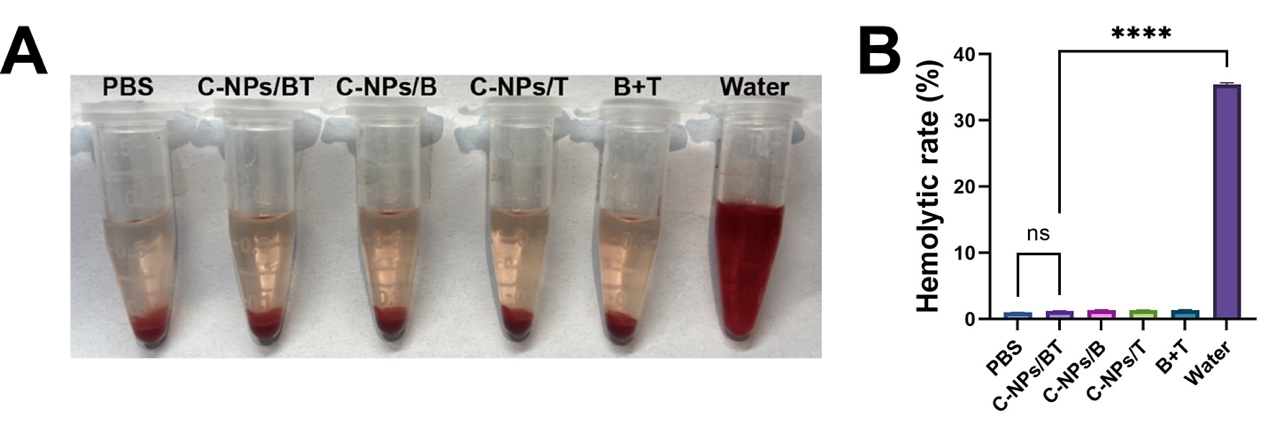


**Figure S3.** Hemolysis test to test the safety of preparations. Data was expressed as mean ± SD. No significant difference (ns): P> 0.05 and ****P < 0.0001.


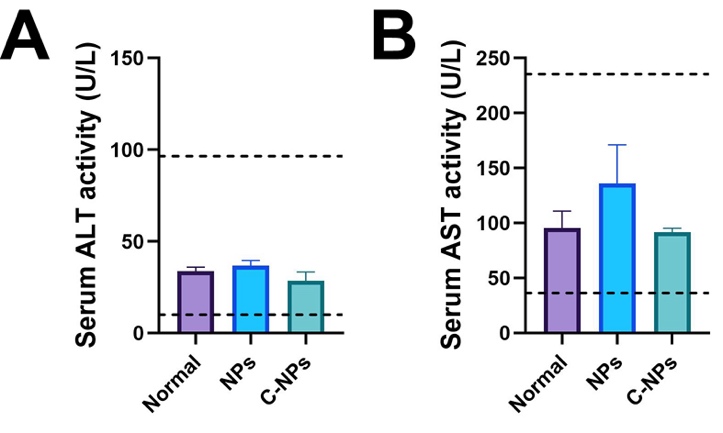


**Figure S4.** Serum A) ALT and B) AST activity of different treatment groups (the dotted line means the normal range). Data was expressed as mean ± SD.


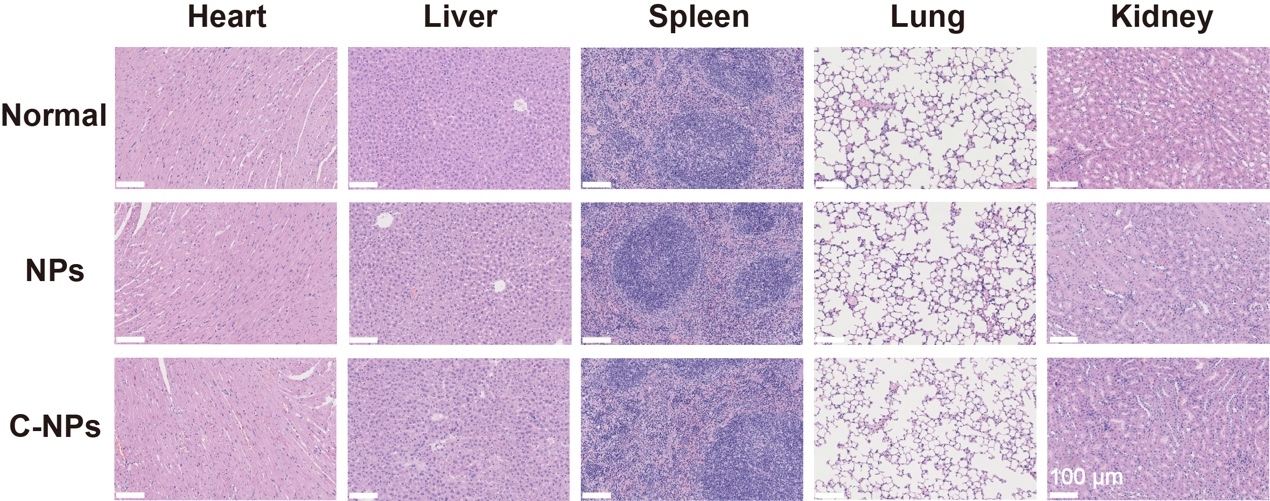


**Figure S5.** H&E staining (200×) of heart, liver, spleen, lung and kidney tissues from normal mice, NPs and C-NPs treated mice.


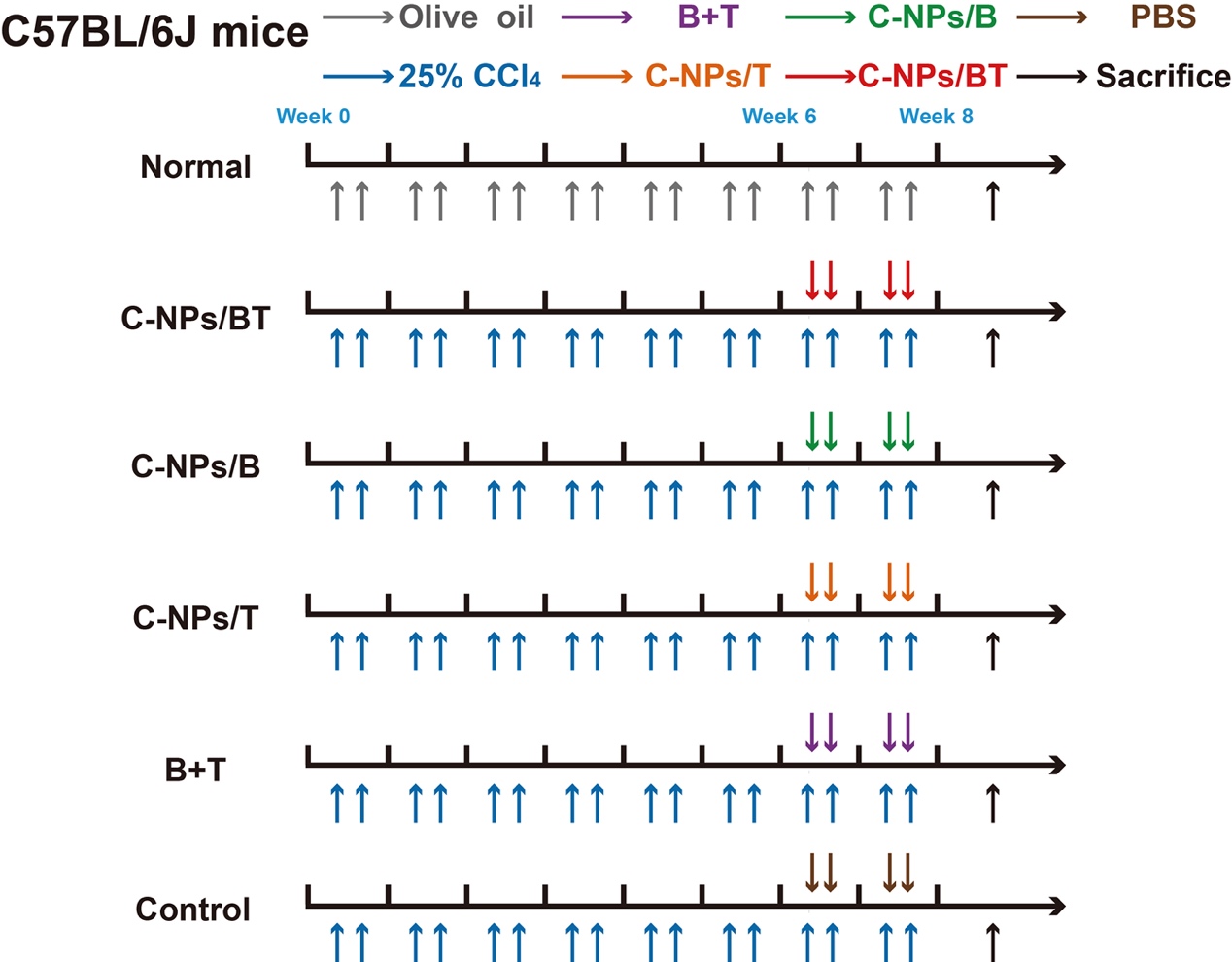


**Figure S6.** Schematic diagram showing the induction of hepatic fibrosis by CCl_4_ in mice, and administration of nanoparticles in different groups.

**
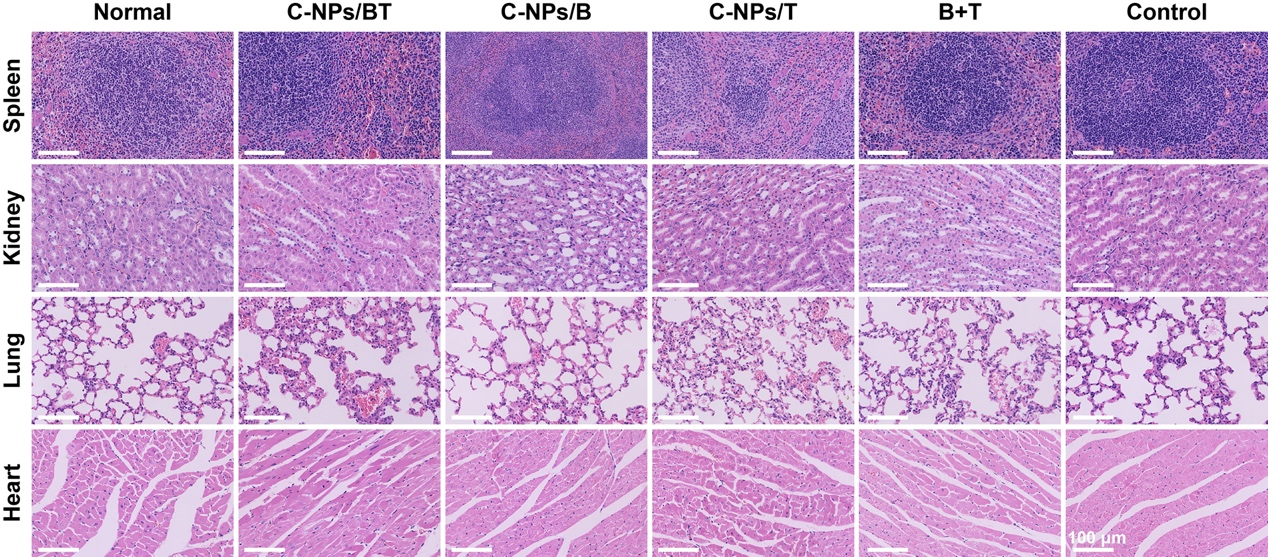
**

**Figure S7.** H&E staining (200×) of spleen, kidneys, lung and heart tissues from different treatment mice.


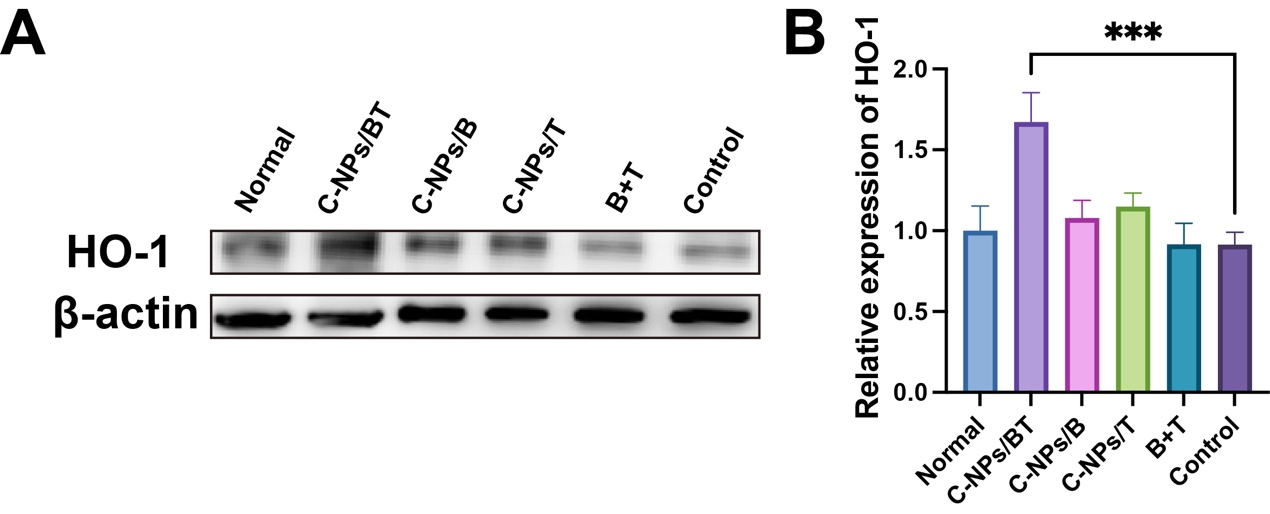


**Figure S8.** Expression of HO-1 in liver tissues. A) Western blot analysis of HO-1. B) Semi-quantification of HO-1 (n = 3). Data was expressed as mean ± SD. ***P < 0.001.


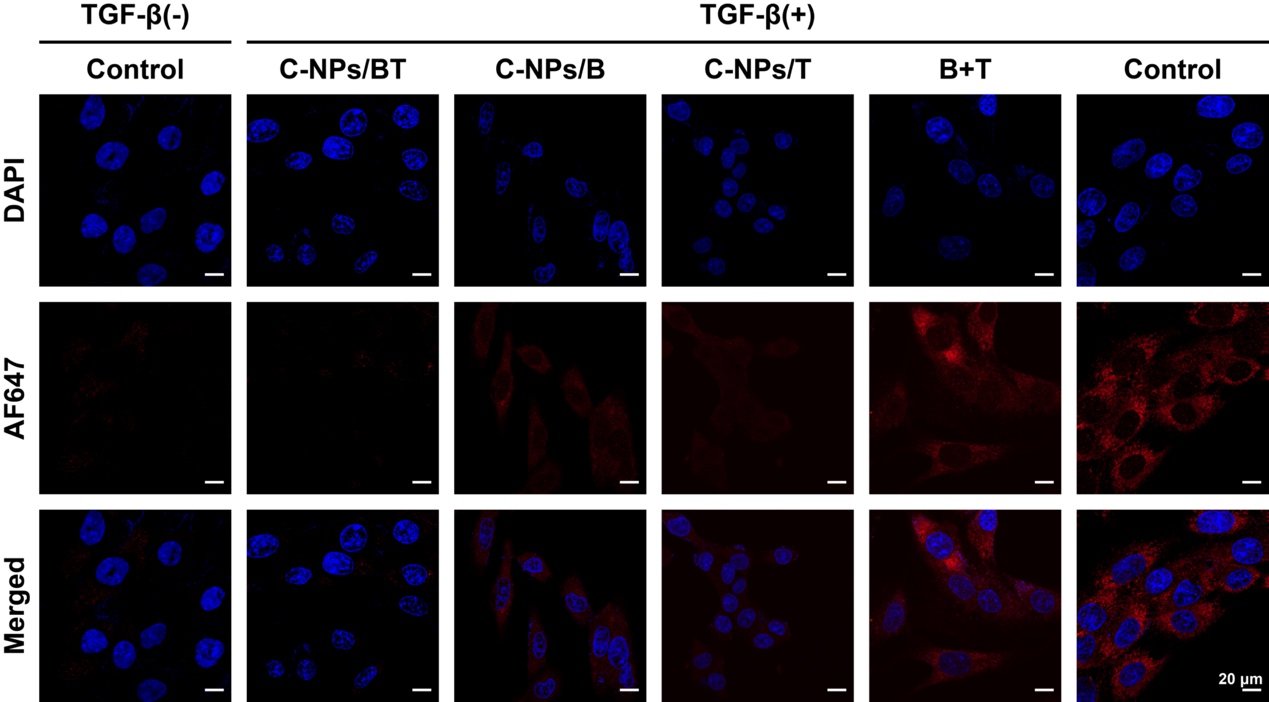


**Figure S9.** Representative images of collagen I in different treatment groups. Scale bar = 20 μm


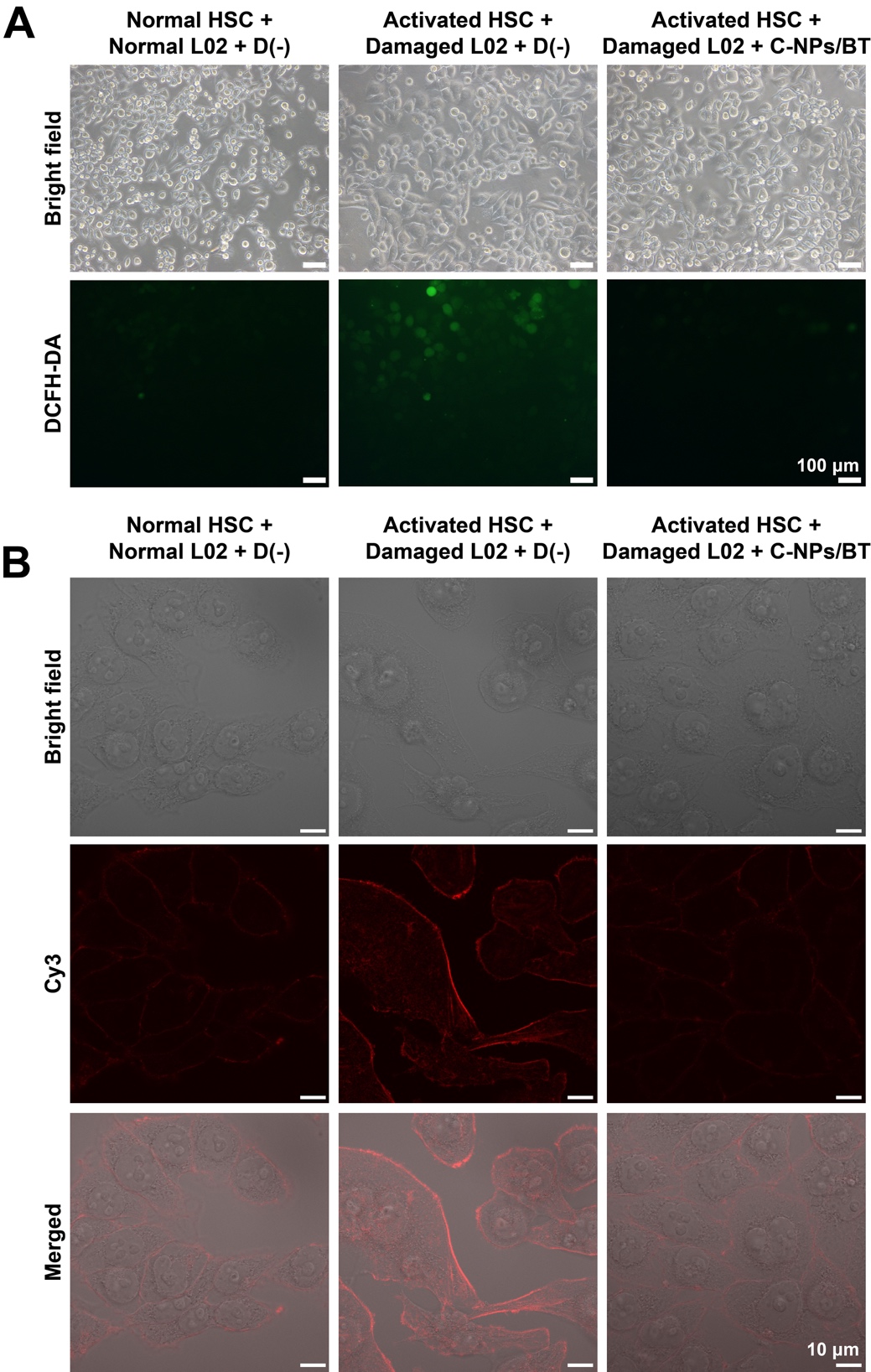


**Figure S10.** HSC-hepatocytes co-culture model. A) Representative images of DCFH-DA. Scale bar = 100 μm B) Representative images of α-SMA. Scale bar = 10 μm
